# Research Advances on Fiber-Optic SPR Sensors with Temperature Self-Compensation

**DOI:** 10.3390/s23020644

**Published:** 2023-01-06

**Authors:** Hongxia Zhao, Feng Wang, Zhaojia Han, Peihong Cheng, Zhiqun Ding

**Affiliations:** 1 Electronic and Information Engineering College, Ningbo University of Technology, Ningbo 315016, China; 2 Faculty of Humanities and Social Sciences, University of Nottingham Ningbo China, Ningbo 315100, China; 3 State Key Laboratory of Silicon Materials, Zhejiang University, Hangzhou 310027, China

**Keywords:** optical fiber sensor, surface plasmon resonance, temperature self-compensation

## Abstract

The fiber-optic surface plasmon resonance sensor has very promising applications in environmental monitoring, biochemical sensing, and medical diagnosis, due to the superiority of high sensitivity and novel label-free microstructure. However, the influence of ambient temperature is inevitable in practical sensing applications, and even the higher the sensitivity, the greater the influence. Therefore, how to eliminate temperature interference in the sensing process has become one of the hot issues of this research field in recent years, and some accomplishments have been achieved. This paper mainly reviews the research results on temperature self-compensating fiber-optic surface plasmon sensors. Firstly, it introduces the mechanism of a temperature self-compensating fiber-optic surface plasmon resonance sensor. Then, the latest development of temperature self-compensated sensor is reviewed from the perspective of various fiber-optic sensing structures. Finally, this paper discusses the most recent applications and development prospects of temperature self-compensated fiber-optic surface plasmon resonance sensors.

## 1. Introduction

Surface plasmon resonance (SPR) is a physical phenomenon that occurs when a P-polarized light wave is incident on the metal-dielectric interface, causing the resonance of free electrons on the metal surface and the absorption of energy [1]. In other words, the SPR characteristic absorption spectrum varies with the physical parameters of ambient refractive index (RI) and temperature on the metal surface. Therefore, the changes in physical quantities such as refractive index and temperature can be inverted by the drift of the SPR spectrum, and then the parametric detection can be accomplished.

The fiber optic SPR sensor is a brand-new type of sensor that combines the advantages of both fiber optic and SPR sensing technologies [2]. In addition to the inherent advantages of fiber optic sensor, the SPR sensor has the additional advantages of high detection sensitivity and label-free detection. This technology has been widely used in physical quantity measurement [3,4,5,6,7], biological quantity sensing [8,9,10,11,12,13,14,15,16], and environmental monitoring [17,18,19]. However, temperature variation affects the refractive index of metals and dielectric materials, and according to the SPR resonance wavelength calculation equation [20], $\lambda_{\mathrm{sp}}=\lambda_{0}\sqrt{\frac{1}{\varepsilon_{m}}+\frac{1}{\varepsilon_{d}}}$ ($\lambda_{0}$ is the incident wavelength, and $\varepsilon_{m}$ and $\varepsilon_{d}$ are dielectric constants of the metal film and ambient medium, respectively), the resonance peak wavelength shift of the SPR due to temperature change is unavoidable. As a result, compensating for temperature noise is a necessary consideration when performing sensing demodulation of non-temperature to be measured based on the resonant wavelength of the SPR resonance spectra. In particular, the influence of ambient temperature cannot be avoided, and the smaller the fiber cladding diameter, the larger the influence of temperature. As a result, the implementation of temperature self-compensating in high-sensitivity sensing applications has become critical to the practical application of fiber-optic SPR sensors.

The research on structural design, experimental setup, and application of temperature self-compensating fiber-optic SPR sensor has advanced greatly due to the booming development of correlation technology. For example, using the MMF-SMF-MMF sandwich structure, Wang et al. [20] vaporized Au film on the SMF surface while coating PDMS, a temperature-sensitive material, on half of the length of the surface to excite concentration and temperature dual SPR channels, respectively, and performed temperature-compensable glucose concentration measurements. In addition, Teng et al. [21] obtained temperature-compensated refractive index measurements with a sensitivity of up to 1258 nm/RIU by vaporizing Au films on both polished sides of a U-shape plastic fiber and then coating one of the Au film surfaces with PDMS. Lia et al. [22] coated PDNA and PH-sensitive material PAA/CS on the surface of FBG, eliminated the influence of ambient temperature and ph value and realized DNA sequence detection. To excite temperature-sensitive SPR channels, Wei et al. [23] used a graded-index multi-mode fiber (GI-MMF) as the displacement sensing region (DSR) and hetero-core fiber as the temperature sensing region (TSR) coated with polydimethylsiloxane (PDMS) temperature-sensitive material. They also investigated micro-displacement sensing, which can be realized with temperature compensation by changing the radial position of the single-mode fiber (SMF). However, there is no review paper on the pertinent aspects of such a sensor so far. This paper conducts a review study specifically for fiber optic SPR sensors with temperature self-compensation based on the review of related literature. It primarily introduces the mechanism, various fiber optic micro-/nano-structures for temperature self-compensating sensing, and the most recent applications.

## 2. The Mechanism of Temperature Self-Compensating Fiber-Optic Surface Plasmon Resonance Sensors

**Figure 1:**
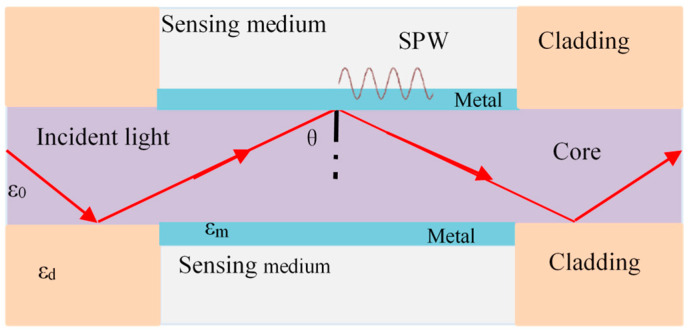
Schematic diagram of optical fiber SPR sensor.

Figure 1 depicts the typical three-layer construction of a fiber-optic SPR sensor: the sensing medium, the metal, and the core [24]. The dielectric constants of the core, the metal, and the sensing medium are, respectively, represented by *ε*_0_, *ε*_m,_ and *ε*_d_. When incident light is transmitted in the fiber core at a specific angle, the light wave is completely reflected at the interface between the core and the metal layer. However, some of the energy will enter the metal layer as an evanescent wave, exciting the surface plasma resonance wave. Typically, the transmission constant of a plasma wave on a metal surface can be expressed as [25]:

(1) $$K_{\mathrm{sp}}=\frac{\omega }{c}\sqrt{\frac{\varepsilon_{m}\varepsilon_{d}}{\varepsilon_{m}+\varepsilon_{d}}}$$

where *c* is the speed of light in a vacuum, and *ω* is the angular frequency of the incident light wave.

According to Figure 1, the propagation constant of an evanescent wave, that is, the parallel component of the interface between metal and core media is expressed as [26]

(2) $$K=\frac{\omega }{c}\sqrt{\varepsilon_{0}}\sin(\theta )$$

where *θ* is the incident angle. When the incident angle or incident wavelength is an appropriate value, the propagation constants of evanescent wave and plasma waves meet the phase matching conditions, which means *K*sp = *K*. At this moment, the surface plasma wave and the evanescent wave interact resonantly. The portion of incident light wave energy that meets the resonance criteria is converted into the oscillation energy of plasma wave, which weakens the reflected light and finally presents a resonance absorption peak in the transmission spectrum. According to [27], the optical power of the resonance absorption peak is given by

(3) $$P_{t}=\frac{\int_{\theta_{C}}^{\frac{\pi }{2}}R{\left(\theta \right)}^{N(\theta )}I(\theta )d\theta }{\int_{\theta_{C}}^{\frac{\pi }{2}}I(\theta )d\theta }$$

where N(θ) indicates the number of times light is reflected through the sensing area, $\theta_{c}$ is the critical angle of the light wave at the fiber core–metal interface, I(θ) is the emitted optical power, and *R*(*θ*) denotes the reflectivity of the light wave after passing through the three-layer sensing head, which is related to the refractive index of the medium and the incident angle of the light wave. *ε*_d_ and *ε*_m_ are the physical variables of temperature and pressure. Therefore, cross-sensitivity exists in practical sensing applications, and the influence of temperature is unavoidable, especially under non-isothermal conditions. When the mixed sensing sensitivity is known, the sensitivity matrix equation can be expressed as

(4) $$\left[\begin{array}{c} \Delta T\\ \Delta N \end{array}\right]=\frac{1}{F}\left[\begin{array}{cc} T_{\lambda 1} & T_{\lambda 2}\\ N_{\lambda 1} & N_{\lambda 2} \end{array}\right]\left[\begin{array}{c} \Delta \lambda_{1}\\ \Delta \lambda_{2} \end{array}\right]$$

where Δ*T* and Δ*N* are temperature and the change to be measured, Δ*λ*_1_ and Δ*λ*_2_ are the drift of the sensing channel’s wavelength, and *T_λ_*_1_, *T_λ_*_2_, *N_λ_*_1_, and *N_λ_*_2_ are the sensing sensitivity of the sensing channel to the temperature and the parameter to be measured. $F=T_{\lambda 1}N_{\lambda 2}-N_{\lambda 1}T_{\lambda 2}$. With the help of (4), the refractive index sensing sensitivity that eliminates the effect of temperature can be obtained [28,29,30,31].

## 3. The Structure of Temperature Self-Compensating Fiber SPR Sensors

**Figure 2:**
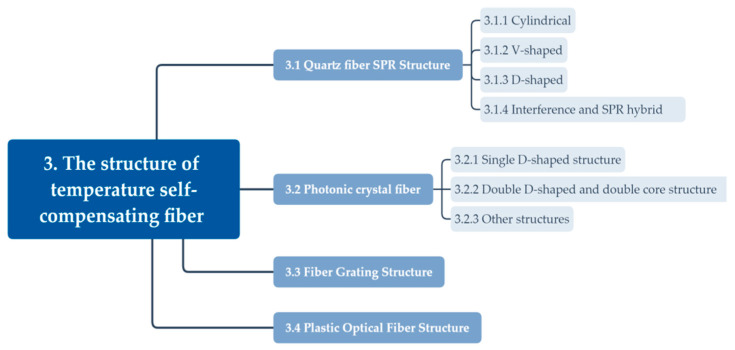
The overall structure of the temperature self-compensating fiber SPR sensor.

The main contents of the structure of temperature self-compensating fiber SPR sensors are summarized in Figure 2.

### 3.1. Quartz Fiber Surface Plasmon Resonance Microstructure

Quartz fiber is a type of circular wave-guide structure with a core and cladding, which is made of high refractive pure quartz doped with various impurities, such as multi-mode optical fiber (MMF), single-mode optical fiber (SMF), hollow fiber (HF), and no-core fiber (NCF). Temperature self-compensated single-parameter or multi-parameter sensing can be achieved by removing different parts and shapes of the cladding by modern micro-nano fabrication technology, polishing and vaporizing metal nano-films such as Au and Ag, and coating them with high thermo-optical coefficient materials. This sensor has simple manufacturing and high mechanical strength. The following are the reported design structures.

#### 3.1.1. Cylindrical Structure

**Figure 3:**
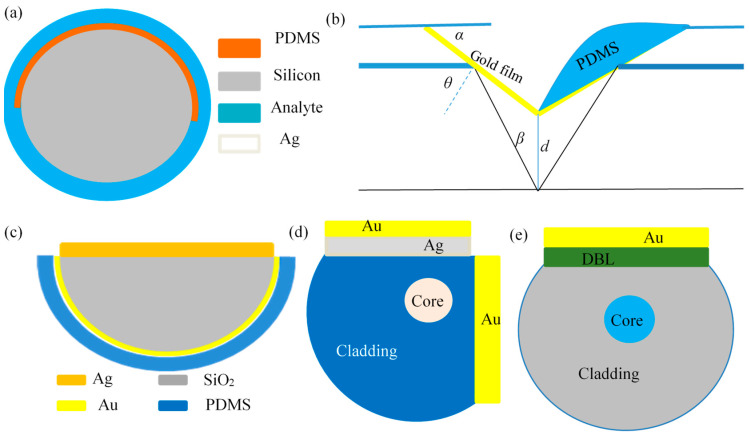
Quartz fiber SPR structure. (**a**) Cylindrical [36]; (**b**) V-shaped [38]; (**c**) D-shaped [39]; (**d**) Vertical double-D [42]; (**e**) Long-range SPR [44].

It is based on the difference of a dielectric constant of the evanescent wave field at different areas, which excites the dual SPR resonant modes and achieves dual-parameter without any further fiber structure processing. This is accomplished by evaporating a metal film on the surface of fiber cladding at a certain length and then spin-coating a temperature-sensitive material. Double SPR resonant modes are excited based on the difference of dielectric constants for different parts of the evanescent wave field, and double-parameter demodulation is realized. Li, Alonso-Murias [32,33], and Velázquez-González et al. [34] sputtered a 30–50 nm thick gold film on the surface of a 2 cm-long SMF connected to a multimode fiber and then spin-coated a temperature-sensitive material, polydimethylsiloxane (PDMS), on the surface or half of the gold film. By demodulating the reflected or transmitted signals, they were able to achieve refractive index and salinity sensitivities of 2664.540 nm/RIU and 0.31 nm/% which are free from temperature interference. Zeng et al. [35] sputtered Ag and Au nano-films on the inner and outer surfaces of a 2 cm-long HF, respectively, and applied PDMS on the Ag film surface, which achieved refractive index sensing without temperature effects. In addition, Yin et al. [36] obtained a sensing sensitivity of up to 5200 nm/RIU after eliminating the effect of temperature by coating a silver film on the surface of an NCF and applying PDMS on the surface of the semicircular silver film, as shown in Figure 3a. In addition, Li et al. [37] coated the surface of a 1 cm-long NCF with a gold film, followed by coating half the area of the gold film with PDMS and the rest with a silver film. The sensitivity of the glucose concentration sensor was 2.882 nm/% and free from temperature effects.

#### 3.1.2. V-Shaped Structure

Liu et al. [38] machined a homemade eccentric 28 μm quartz fiber into a V-shape; the upslope surface is coated by the gold film to excite SPR resonance absorption peaks for refractive index sensing, while the downslope surface is coated by gold film and then PDMS to obtain the temperature sensing channel. As shown in Figure 3b, the temperature-compensated refractive index sensing sensitivity can reach 3376 nm/RIU in the refractive index range of 1.333 to 1.385. However, the V-shaped slope is difficult to construct and use in real practice.

#### 3.1.3. D-Shaped Structure

This structure is realized by grinding or etching a portion of the cladding within 1 cm to 2 cm along the flank of the fiber, which can improve the interaction between the evanescent wave field and the outside. It is the preferred structure for a fiber SPR sensor due to the easier flat coating process compared to a cylindrical structure. Li et al. [39] ground the 1 cm region of an NCF into a D-shape and then coated the surface with a silver film to excite the SPR A channel. In the meantime, they coated the bottom circular surface with a 50 nm gold film and then cured it with a thin PDMS layer to excite the temperature-sensitive channel B. As shown in Figure 3c, the refractive index sensing sensitivity after temperature compensation was as high as 12,530 nm/RIU in the range of 1.33 to 1.44.

Liu et al. [40] coated a 50 nm gold film on the surface of a D-shaped SMFfollowed by PDMS in half of the area of the gold film. The refractive index sensing sensitivity after temperature compensation is 6300 nm/RIU. Cai et al. [41] ground the upper and lower sides of the fiber into a double D-shaped surface, and sputtered Ag and Au micro-nano films respectively to obtain a refractive index sensing sensitivity of 2441 nm/RIU after temperature compensation; Weng et al. [42] ground the MMF into a D-shaped plane in the horizontal and vertical directions and coated Au and Ag double films, respectively, as shown in Figure 3d. The refractive index and temperature sensing properties of the SPR resonant dual channel were investigated theoretically and experimentally.

Usually, the transmission distance of the SPR mode is only equal to the wavelength of the incident light, and the depth of the SPR absorption peak is not large, around 100–150 nm [43]. In order to increase the transmission distance and depth of the SPR absorption peak, Wang et al. [44] designed a four-layer structure of a fiber core/ dielectric buffer layer/ Au film/ test object, as shown in Figure 3e. With this structure, not only can the interference of temperature be eliminated, but it can also increase the Q factor to as high as 67.75 RIU −1.

#### 3.1.4. Interference and SPR Hybrid Structure

**Figure 4:**
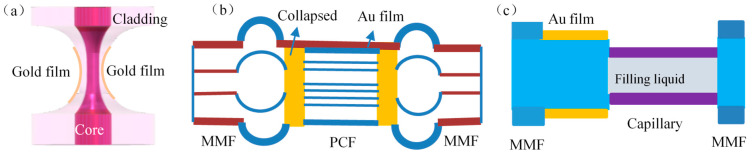
Interferometric Hybrid SPR Structure. (**a**) Tapered structure [45]; (**b**) PCF structure [46]; (**c**) Capillary structure [47].

In order to eliminate the effect of temperature, it is a common and simple idea to construct a temperature-sensitive interference channel. Chen et al. [45] constructed a tapered structure using an SMF, as shown in Figure 4a, to form an MZI interference temperature sensing channel and deposited an Au film on the surface of the tapered waist to excite the SPR absorption mode. The refractive index sensitivity free from temperature effects is up to 2021.07 nm/RIU. 

Tong et al. [46] connected a 1 cm-long PCF between two MMFs and coated Au film on the surface of the PCF to build MZI and SPR dual channels, as shown in Figure 4b. The refractive index sensing after eliminating the temperature effects can reach 1947.405 nm/RIU. At the same time, Chen et al. [47] achieved dual-parameter sensing of Con A and RNA enzyme by constructing F–P interferometry thermo-sensitive channels by connecting capillaries between two MMFs, and depositing Au films on the surface of one of the MMFs to excite SPR resonant channels, as shown in Figure 4c.

### 3.2. Photonic Crystal Fiber Structure

Photonic Crystal Fiber (PCF), also known as holey optical fiber (HOF) or micro-structured optical fiber (MOF), is a silica fiber in which cladding is arranged in an air hole structure regularly along the fiber axis. Because the shape and position of the core and the structure of the air holes in the cladding can be flexibly designed, the direct coupling resonance mode can be generated by changing the shape and position of the air holes, and the filling material of the air holes. In addition, when the surface of the fiber or within the inside of holes is evaporated by metal micro-nano film to excite SPR resonance, the parametric sensing application of eliminating temperature interference can be realized, which has become a hot topic of scientific research in recent years and has made great progress. At present, the reported functional PCF-SPR temperature self-compensation sensor structures mainly include: 

#### 3.2.1. Single D-Shaped Structure [48,49,50,51,52,53]

**Figure 5:**
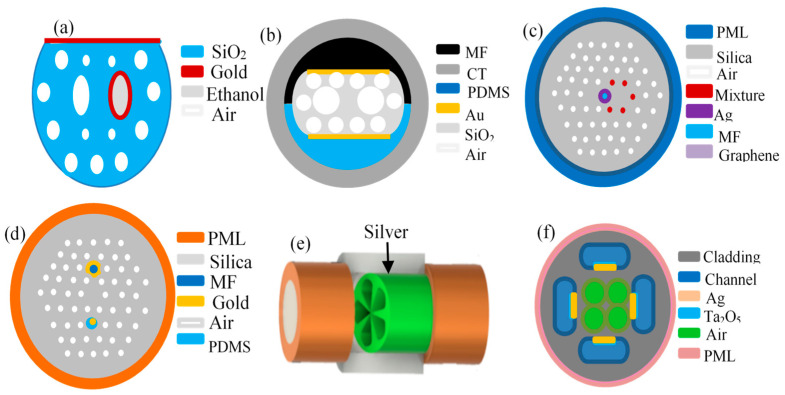
Structure of photonic crystal fiber. (**a**) Single D-shaped structure [49]; (**b**) Double D-shaped [55]; (**c**) double core-shaped structure [57]; (**d**) dual-polarized modes [58]; (**e**) MMF-MOF-MMF structure [59]; (**f**) Large-small hole filling structure [60]. CT, Capillary tube; PML, Perfect match layer.

This type of structure is to coat gold or silver film on the surface of D-shaped PCF with partial cladding removed, exciting the SPR resonance absorption peak. In the meantime, an air hole is selected by different position, shape, and size to fill with high thermo-optic coefficient liquid, such as chloroform, PMDS, ethanol, or toluene, to sense the change of ambient temperature. As shown in Figure 5a, Chen et al. [49] sputtered the surface of D-shaped PCF with gold film to excite the SPR channel for RI sensing and filled the air hole near the fiber core with temperature-sensitive liquid ethanol after gold coating to sense the temperature.

#### 3.2.2. Double D-Shaped [54,55] and Double Core Structure [56,57]

The double D-shaped structure is to remove part of the cladding by polishing the two sides of PCF to D-shaped planes, then coat the planes with metal nanofilm, and fill PDMS at the location of one of the metal film. Then, dual SPR channels at the two sides are motivated to achieve temperature self-compensated measurements according to the differential sensitivity of the dual SPR channels to temperature and magnetic field, as in Figure 5b. Figure 5c is the structure of a double-core PCF sensor head. The center air hole between the two cores is coated with gold film and covered with a single layer of Graphene, and then injected with magnetic fluid (MF). The five holes on the right side are filled with a mixture of chloroform and toluene to excite the temperature-sensitive channel, which realizes the magnetic-field intensity sensing measurement free from temperature effects.

#### 3.2.3. Other Structures

Liu et al. [58] coated gold film in the large air holes and filled them with MF, while the small air holes were filled with PDMS and gold wires, which also realized the magnetic-field intensity sensing measurement free from temperature effects, as shown in Figure 5d. Wang et al. [59] connected a large core MMF to POF, where the surface of POF was coated with Ag film; then, the SPR channel under test was activated. Due to the air hole from the collapse effect in the MMF-POF welding process, the temperature sensitivity of SPR was weak and the effect of temperature was naturally eliminated, as shown in Figure 5e. Gu et al. [60] set up four microfluidic cavities in the fiber cladding, as shown in Figure 5f. Ag-Ti_2_O_5_ was evaporated in turn close to the fiber core, the upper and lower microcavities were filled with MF, and the left and right microcavities were injected with temperature-sensitive materials to excite the orthogonal SPR mode. This structure also achieves magnetic field measurement that eliminates the effect of temperature.

The PCF microstructure sensors can realize temperature self-compensation multi-parameter sensing based on SPR resonant mode and direct coupled mode. However, the production equipment is expensive, most of the research is limited to theoretical simulation, and the experimental preparation research is less. We believe that the technology will be desirable in practical application with the popularization of laser direct writing equipment and the continuous improvement of the preparation process. 

### 3.3. Fiber Grating Structure

**Figure 6:**
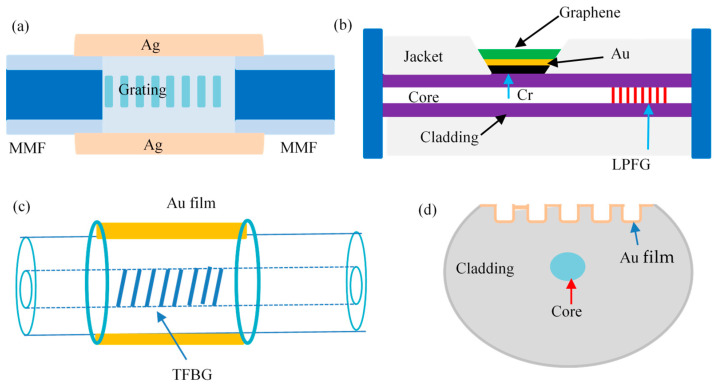
The structure of fiber grating sensing head (**a**) Bragg grating structure [61]; (**b**) long-period fiber grating structure [62]; (**c**) tilted fiber grating structure [63]; (**d**) surface grating structure [64].

The fiber grating is a coupling structure formed by modulating the refractive index of the fiber core axially and periodically by certain methods, which mainly includes the Bragg fiber grating coupled with the incident light wave, the tilted fiber grating coupled with the reverse light, and the long-period fiber grating based on coupling between isotropic transmission core mode and the cladding mode. The combination of fiber grating and SPR technology is an all-fiber structure with the advantages of small size, high sensitivity, and multi-point distributed sensing. Furthermore, the resonant wavelength is sensitive to temperature and plays an outstanding role in temperature self-compensation SPR sensing technology. Hu et al. [61] designed an all-fiber structure of MMF-FBG-MMF as shown in Figure 6a. The temperature self-compensation RI sensor is realized based on the different sensitivity of double resonance wavelength to temperature and RI by depositing a silver film on the cladding surface of FBG to excite SPR. Figure 6b is a temperature self-compensating glucose concentration sensor based on LPFG designed by Lu et al. [62]. The SPR resonant absorption peak is excited by sputtering a certain nanometer thickness of Cr and Au films on the surface of SMF, and then coating a single layer of Graphene to improve the sensing sensitivity. Finally, the borate polymer PAA-ran-PAAPBA was used as an adsorbent to realize the specific detection of glucose concentration. 

Figure 6c shows the temperature self-compensation composite refractive index sensor based on tilted fiber grating and SPR technology proposed by Shao et al. [63]. In addition, Yu et al. [64] designed a temperature self-compensated refractive index sensor with a silicon core surface grating structure, as shown in Figure 6d. The surface grating is obtained by photolithography of silicon core fibers after side polishing, and the SPR resonance absorption peak is excited by plating a 2 nm gold film on the surface of the grating. Then, a double peak is generated based on the coupling between the core and SPR modes with different refractive indices in the X and Y polarization directions. With the help of the different sensitivity of the double peak to temperature and refractive index, an ethanol concentration measurement free from temperature interference is realized.

### 3.4. Plastic Optical Fiber Structure

Quartz optical fiber sensor has been studied for a long time. In 1970, the Corning Company successfully developed low-loss optical fiber, and remarkable achievements have been made in the structural design [65,66,67], experimental preparation [68,69,70,71,72], and application [73,74,75,76,77] of the sensor head. However, in order to solve the cross-sensitivity problem of quartz optic fiber sensors, structural modification is required, which leads to the sensor head being brittle and easy to break. Then, it is limited in some special environments. Thus, in recent years, researchers have again focused on plastic optical fibers.

Plastic Optical Fiber (POF) is a class of waveguide structures with highly transparent polymers as fiber cores and cladding material. It has the advantages of being lightweight, and having softness, low modulus, a large core diameter (0.3–1.0 mm), and so on. The good deflection can reach the sub-millimeter scale, and it has excellent tensile strength, simple preparation, and processing operation, being especially suitable for bending occasions and rough environments. In 2021, Liu et al. coated the polished surfaces of symmetrical D-shaped POF with a gold film, and vertically sprayed PDMS on one side of the gold film surfaces to obtain SPR bimodal sensing channels [78]. The high sensitivity refractive index sensing is realized after the temperature interference is eliminated.

Although the POF sensor is still in the initial stage, the vast application prospect is predictable due to its low loss window in the visible light band, the strong sensitivity of light intensity to humidity, chemicals, and solutions, as well as a wide selection of materials.

In order to provide a certain reference for the researchers to develop better performance fiber SPR sensors with Temperature Self-Compensation, we compare and analyze the methods and summarize the advantages and disadvantages, as shown in Table 1.

## 4. Application of Temperature Self-Compensating Fiber SPR Sensor

### 4.1. Environmental Monitoring

**Figure 7:**
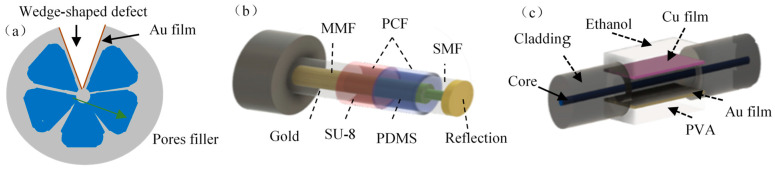
(**a**) a six-wedge-hole fiber-optic sensing structure [82]; (**b**) a reflective three-parameter sensor [85]; (**c**) a dual-D humidity sensor [86].

Seawater salinity is a scale to measure the salt content of seawater, which is a fundamental parameter in the study of the physical and chemical processes of seawater. The accurate detection of seawater salinity and its distribution in the ocean has very important practical value in the fields of oceanographic research, monitoring and prediction of marine climate and environment [79], marine military [80], coastal oil production, and marine fishery [81]. Zhao et al. [82] removed a wedge-shaped hole from the six-wedge fiber, as shown in Figure 7a, to introduce birefringence in both X and Y directions. The inner surface of the defect was plated with Au, and the thermo-optic material was filled into the air hole. Dual SPR resonance loss peaks excited in X and Y directions are used to sense salinity and temperature simultaneously, and the sensitivity value of salinity sensing after temperature compensation is 1.402/%. In order to further improve the salinity sensing sensitivity, Yang et al. [83,84] sputtered two-layer films of ITO and Ag in turn at the defect of the six-wedge fiber to form the Kretschmann and Otto models, which excite the double SPR modes, respectively. Based on the large tunable range of the refractive index of ITO, the salinity sensing sensitivity free from temperature effects is increased to 4.45 nm/%.

Zhao et al. constructed an MMF-PCF-SMF reflective composite fiber sensing head [85], as shown in Figure 7b. In order to excite multiple resonant SPR absorption peaks, a PCF optical fiber is set in the middle of the sensor head as a multi-mode excitation field, and the different areas of the PCF’s surface are coated by temperature-sensitive material PMDS and pressure-sensitive material SU-8, respectively. Three SPR modes can be excited simultaneously for the sensing of salinity, temperature, and pressure of seawater, respectively. The sensitivity matrix equation is used to obtain the salinity and pressure values that eliminate temperature disturbances.

Wang et al. coated Au film and Cu film on the upper and lower surfaces of the symmetrical double D-shaped MMF [86], respectively, and the surface of Cu film was painted with ethanol, while the surface of Au film was painted with polyvinyl alcohol, as shown in Figure 7c. Due to the differential sensitivity of ethanol and polyvinyl alcohol to temperature and humidity, the humidity sensing sensitivity is up to 11.6 nm/%RH in the range of 20–80 %RH.

### 4.2. Physical Quantity Sensing

In essence, the fiber-based SPR sensor is only sensitive to the refractive index of the medium to be measured. However, it has been widely used to sense physical quantities such as stress, pressure, curvature, and concentration, which is due to the help of sputtering metal film on the surface of optical fiber, and modifying functional auxiliary film on the film surface or combining with new fibers such as photonic crystal fiber, hollow fiber, and coreless fiber. Furthermore, the sensing sensitivity can be improved by eliminating the interference of ambient temperature through structural or material design.

**Figure 8:**
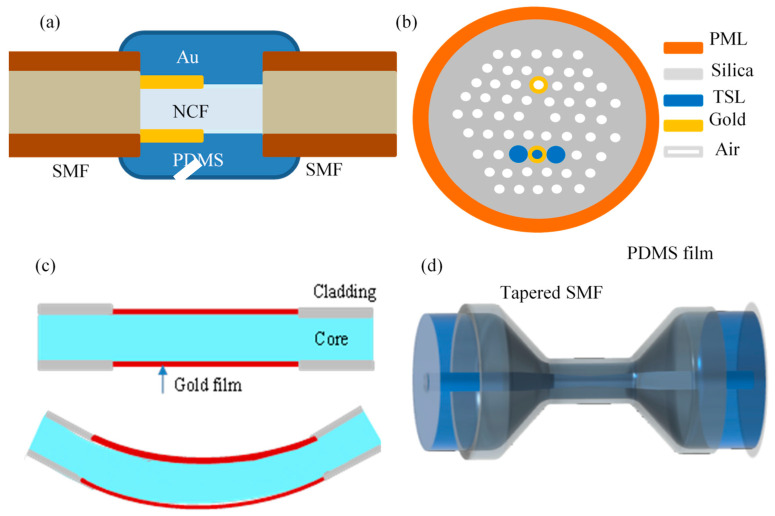
Physical sensor structure. (**a**) no-core fiber optic strain sensing [87]; (**b**) photonic fiber optic strain sensing [88]; (**c**) bend sensing [89]; (**d**) Pressure sensing [90]. PML, **Perfect match layer; TSL, Temperature Sensitive Liquid.**

Duo et al. constructed an interferometric fiber-based SPR sensor by sequentially connecting SMF, NCF, and SMF [87], as shown in Figure 8a. A 30 nm Au film was evaporated on half of the surface of the NCF, and then the whole surface of the NCF is wrapped with PDMS to excite SPR resonant mode for the sensing of ambient temperature. Furthermore, the multimode interference spectrum formed in NCF was used to sense stress. Finally, the stress sensing sensitivity, which eliminates the interference of ambient temperature, up to 54.1 pm/με is obtained by using resonant wavelength and normalized light intensity demodulation. In the same year, Han et al. also achieved the simultaneous measurement of strain and temperature with PCF fiber [88], as shown in Figure 8b. Two symmetrical air holes in the cladding layer are coated with gold, and one of the gold-plated air holes is filled with diethylene glycol temperature-sensitive material. Then, two SPRs are excited simultaneously to demodulate strain and temperature based on dual resonance absorption wavelengths. Su et al. constructed a 1.5 cm-long three-layer typical SPR structure using MMF [89]. The thickness of the Au film is preferably 45 nm, as shown in Figure 8c. The curvature change free from the temperature effect is achieved by two parametric demodulation methods with the single SPR resonance absorption peak wavelength and light intensity. The experimental results prove that the increase of fiber curvature and decreasing of resonant angle result in the redshift of SPR resonant absorption peak wavelength, and the enhancement of evanescent wave results in the weakening of SPR peak transmittance. In addition, with the increasing temperature, the SPR peak has a blue shift, and the transmittance increased. Figure 8d shows the structure of the pressure sensor with micron-scale PDMS on the surface of the quartz single-tapered fiber proposed by Zhao [90]. Due to the large aperture of PDMS, it is suitable for gas pressure sensing. The interference spectrum generated by the single-tapered fiber is used to sense temperature. In the range of 1 KPa-100 KPa, the pressure sensing sensitivity is as high as −159.80 nm/MPa.

**Figure 9:**
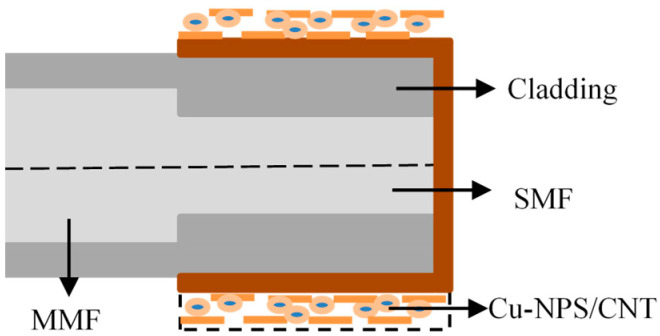
Structural diagram of the nitrate concentration sensor [91].

Zhang et al. constructed a reflective sensing structure by plating Au film on the end and surface of a 1 cm-long SMF connected to an MMF [91]. Under the catalysis of Cu particles and carbon nanotubes on the surface of the gold film, as shown in Figure 9, nitrate reacts with hydrogen ions to generate ammonia ions, which are absorbed by carbon nanotubes to induce a change in the refractive index. It causes the shift of SPR resonance wavelength, which can be used to sense the concentration of nitrate. At the same time, half of the gold film surface is coated with a temperature-sensitive material PDMS to sense the ambient temperature. The sensitivity of the nitrate concentration sensing after temperature compensation reaches 3.25 nm/lg[M].

### 4.3. Biological Detection

Biomass sensing is a technique to detect biological components by observing biological changes such as coupling, reaction, and reconstruction. The interaction of biological macromolecules on the surface of the fiber-based SPR sensor can cause a large change in refractive index, and the sensing structure can directly contact the object to be measured, which has the advantages of fast detection speed and high sensitivity. As a result, fiber-based SPR sensors have become a powerful test tool for biological component analysis and are widely used. 

**Figure 10:**
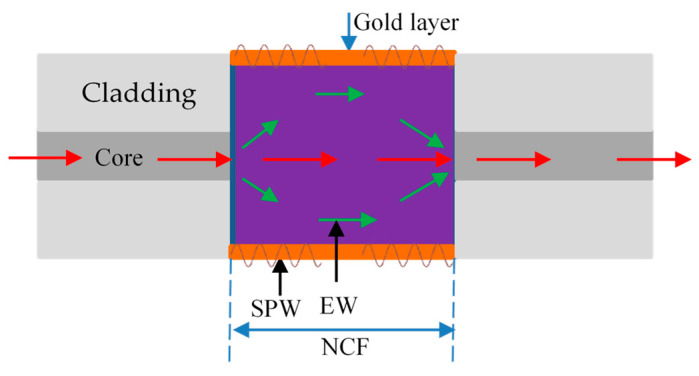
Schematic diagram of the DNA hybridization sensor [92].

Gang et al. designed a temperature self-compensating fiber optic DNA hybridization sensor by combining SPR and MZI technologies [92], as shown in Figure 10. The surface of the 8 mm-long NCF connected to an SMF is coated with Au film and modified with pDNA antibody for specific detection of cDNA. At the same time, the MZI interference resonance peak generated by the coreless part of NCF is used to compensate for the impact of ambient temperature. This method has high sensitivity and broad application prospects for specific DNA detection such as gene diagnosis, criminal investigation, and paternity tests. Lia et al. [22] sputtered Au film on the surface of FBG made of silica, complemented with exon-20 and PAA/CS, eliminated the influence of environmental temperature and pH value, and realized the detection of genetic factors of lung cancer. The sensitivity to exon-20 is 0.04 nm/nM, and the LOD of exon-20 reaches 13.5 nM. 

## 5. Conclusions 

Temperature self-compensating SPR sensors have been studied intensively in recent years, yielding a series of impressive results. Scholars have used a variety of methods to achieve temperature self-compensating measurements, which not only account for the effects of ambient temperature when measuring liquid RI but also broaden the measurement range to include different types of biochemical analytes and even physical parameters such as magnetic fields and salinity. The numerous examples in Section 3 prove that the temperature self-compensating sensing structure not only maintains the advantages of SPR sensors’ fast response and high sensitivity but also makes measurement data more comprehensive and accurate, which has significant research value and broad application prospects. In general, fiber-based SPR sensing with temperature self-compensation has shown attractive promise, and there are still some challenges for this type of sensors: (1) the SPR resonance absorption spectra have a wide bandwidth in the submicron range, which severely limits the measurement sensitivity; (2) the problems of practical use still exist, and further steps should be taken based on the research of sensing mechanisms and structural innovations; (3) the vast majority of fiber-based SPR sensors use wavelength modulation, and the demodulation equipment is relatively expensive, so diverse demodulation methods need to be further developed. Nevertheless, the fiber-based SPR sensing technology is still emerging, and will burst out with new vitality and vigor when combined with new materials, new structures, and new technologies—for example, Bloch surface wave (BSW)/Tamm mode-based fiber optical sensors (including D-shaped fiber and PCF) [93,94], which are analogous to SPR but possess better performance. 

## Figures and Tables

**Table 1:** The advantages and disadvantages of the realization methods of TSC-SPR fiber sensing.

Methods of TSC ^(a)^-SPR		Advantages	Disadvantages	Typical Performance Parameters		Ref.
				Sensitivity	Dynamic Range	
QF ^(b)^-SPR	Cylindrical structure	Easy to manufacture and low cost	Loss of some sensing performance	2664.54 nm/RIU	1.346–1.388	[34]
	V-shaped structure	Narrow full width at half maximum and good stability	Complex preparation process	3376 nm/RIU	1.333–1.385	[38]
	D-shaped structure	High sensitivity and large-scale production	fragile structure	2694.3 nm/RIU 5850 nm/RIU 12530 nm/RIU	1.33–1.38 1.38–1.41 1.41–1.44	[39]
	Interference and SPR hybrid structure	Easy to manufacture and ability to compensate for the interference of environmental factors	Complex measurement system	2021.07 nm/RIU	1.333–1.338	[45]
PCF-SPR	single D-shaped structure	high sensitivity and the ability to detect biochemical reaction	Complex measurement system	10,300 nm/RIU	1.41–1.42	[50]
	Double D-shaped and double core structure	applicability to a variety of SPR sensing structures and multi-parameter sensing	Complex measurement system	1371 nm/RIU	1.33–1.34	[54]
	Other structures	good mechanical strength and reproducibility	narrow application range	3223 nm/RIU	1.3328–1.339	[59]
Fiber Grating Structure		High SNR, good sensor mechanical strength	low sensitivity	571.5 nm/RIU	1.332–1.338	[63]
POF-SPR		Easy preparation process	low SNR and narrow–scale production	1174 nm/RIU	1.335–1.37	[78]

^(a)^ TSC is temperature self-compensating, ^(b)^ QF is Quartz fiber.

## Data Availability

Not applicable.
